# Supplementary material for: Predicting the potential impact of scaling up four pneumonia interventions on under-five pneumonia mortality: A prospective Lives Saved Tool (LiST) analysis for Bangladesh, Chad, and Ethiopia
Source: J Glob Health. 2024 Jan 12;14:04001. doi: 10.7189/jogh.14.04001 (PMC10801440; doi:10.7189/jogh.14.04001)
Supplement: Online Supplementary Document [file jogh-14-04001-s001.pdf]

# Online Supplementary Material

Pfurtscheller T, Lam F, Shah R, Shohel R, Sans MS, Tounaikok N, Hassen A, Berhanu A, Dinkineh B, Berryman E, Habte T, Greenslade L, Nantanda R, Baker K. Predicting the potential impact of scaling up four pneumonia interventions on under-five pneumonia mortality: A prospective Lives Saved Tool (LiST) analysis for Bangladesh, Chad, and Ethiopia. J Glob Health 2024;14:04001.

## Contents

Appendix S1. Baseline data sources 1

Table 1. Bangladesh 1

Table 2. Chad 2

Table 3. Ethiopia 2

# Appendix S1. Baseline data sources

**Table 1. Bangladesh**

| Parameter | Value | Reference year | Source |
|---|---|---|---|
| Under-5 mortality rate (per 1,000 live births) | 29.1 | 2021 | UN IGME |
| Percent of post-neonatal deaths due to pneumonia (%) | 16.66 | 2019 | WHO-MCEE |
| Hib coverage (%) | 98 | 2022 | WHO/UNICEF Immunization Coverage Estimates |
| PCV coverage (%) | 99 | 2022 | WHO/UNICEF Immunization Coverage Estimates |
| Oral antibiotics for pneumonia coverage | 34 | 2017 | Bangladesh Demographic and Health Survey 2017-18. |
| Pox and oxygen for pneumonia coverage (%) | 3.17 | 2021 | Bangladesh DHIS2 |

**Table 2. Chad**

| Parameter | Value | Reference year | Source |
|---|---|---|---|
| Under-5 mortality rate (per 1,000 live births) | 110.05 | 2021 | UN IGME |
| Percent of post-neonatal deaths due to pneumonia (%) | 28.05 | 2019 | WHO-MCEE |
| Hib coverage (%) | 58 | 2022 | WHO/UNICEF Immunization Coverage Estimates |
| PCV coverage (%) | 0 | 2022 | WHO/UNICEF Immunization Coverage Estimates |
| Oral antibiotics for pneumonia coverage | 17.6 | 2019 | UNICEF Multiple Indicator Cluster Survey, 2019 |
| Pox and oxygen for pneumonia coverage (%) | 0.69 | n/a | Mangipudi 2020<br>UNICEF Multiple Indicator Cluster Survey, 2019 |

**Table 3. Ethiopia**

| Parameter | Value | Reference year | Source |
|---|---|---|---|
| Under-5 mortality rate (per 1,000 live births) | 48.71 | 2021 | UN IGME |
| Percent of post-neonatal deaths due to pneumonia (%) | 23.63 | 2019 | WHO-MCEE |
| Hib coverage (%) | 65 | 2022 | WHO/UNICEF Immunization Coverage Estimates |
| PCV coverage (%) | 61 | 2022 | WHO/UNICEF Immunization Coverage Estimates |
| Oral antibiotics for pneumonia coverage | 32 | 2019 | Ethiopia Demographic and Health Survey 2016 |
| Pox and oxygen for pneumonia coverage (%) | 11.25 | n/a | Ethiopia Demographic and Health Survey 2016<br>Ministry of Health Ethiopia. National Medical Oxygen and Pulse Oximetry Scale Up Road Map (2016-2020/21) |

*
